# Strong optomechanical interactions in a sliced photonic crystal nanobeam

**DOI:** 10.1038/srep15974

**Published:** 2015-11-02

**Authors:** Rick Leijssen, Ewold Verhagen

**Affiliations:** 1 Center for Nanophotonics, FOM Institute AMOLF, Science Park 104, 1098 XG, Amsterdam, The Netherlands

## Abstract

Coupling between mechanical and optical degrees of freedom is strongly enhanced by using subwavelength optical mode profiles. We realize an optomechanical system based on a sliced photonic crystal nanobeam, which combines such highly confined optical fields with a low-mass mechanical mode. Analyzing the transduction of motion and effects of radiation pressure we find the system exhibits a photon-phonon coupling rate *g*_0_ /2*π* ≈ 11.5 MHz, exceeding previously reported values by an order of magnitude. We show that the large optomechanical interaction enables detecting thermal motion with detection noise below that at the standard quantum limit, even in broad bandwidth devices, important for both sensor applications as well as measurement-based quantum control.

The motion of a mechanical resonator can be read out with extreme sensitivity in a suitably engineered system whose optical response is affected by the displacement of the resonator. The resultant coupling between optical and mechanical degrees of freedom also gives rise to a radiation pressure force that enables actuation, tuning, damping, and amplification of the resonator, with applications ranging from classical information processing to quantum control of macroscopic objects^1^,^2^. Such control can be established either passively, by employing the intrinsic dynamics of the system^3^,^4^,^5^, or actively, by using the outcome of displacement measurements^6^. Fast, sensitive measurement of nanomechanical displacement can as such be used for optical cooling^6^,^7^, squeezed light generation^8^, quantum non-demolition measurements^9^,^10^ and enhancing sensor bandwidth^11^,^12^.

In a cavity optomechanical system, which has an optical resonance frequency *ω*_c_ that depends on the position of a resonator, both the sensitivity of a displacement measurement and the magnitude of effects caused by radiation pressure forces are governed by two parameters: on the one hand the strength with which acoustic and optical degrees of freedom interact, expressed as the magnitude of the resonator’s influence on the frequency *ω*_c_, and on the other hand the cavity linewidth *κ*. The interaction strength is characterized at the most fundamental level by the vacuum optomechanical coupling rate *g*_0_, as it enters the optomechanical interaction Hamiltonian 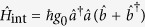, where 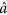 and 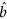 are the photon and phonon annihilation operators, respectively. As this Hamiltonian shows, *g*_0_ describes the frequency response of the optical cavity due to the mechanical displacement in a typical quantum state, where the total number of phonons is of the order of 1.

Per photon in the cavity, the effective optomechanical measurement rate^7^,^8^, as well as the radiation-pressure induced alteration of a resonator’s frequency and damping through dynamical backaction, scale with 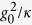. Improving this ratio is thus desirable for more sensitive measurements and for better optical control of the mechanical resonator. Decreasing the optical damping *κ* to a low value has been very fruitful, but can present several drawbacks as well: narrow linewidths place stringent demands on excitation sources and fabrication tolerances, and make integration of many devices, e.g. in practical sensor arrays, difficult. Moreover, dynamical instabilities and nonlinear linewidth broadening limit the number of photons with which a high-Q cavity can be populated. Finally, several schemes for measurement and control in fact rely on fast, broadband optical response^2^,^13^,^14^. The photon-phonon coupling rate *g*_0_, vice versa, is given by *g*_0_ = *Gx*_zpf_, where *G* = ∂*ω*_c_/∂*x* is the frequency shift per unit displacement *x* and 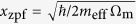 are the zero-point fluctuations of a resonator with mass *m*_eff_ and frequency Ω_m_. The magnitude of *g*_0_ is maximized in suitably engineered miniature systems, as *G* and *x*_zpf_ benefit from small cavity size and small resonator mass, respectively. Indeed, the highest values of *g*_0_ to date have been achieved in micrometer-size devices such as photonic crystal cavities^4^,^8^,^15^,^16^,^17^,^18^ or disk resonators^19^,^20^, with reported values ranging up to about *g*_0_/2*π* ≈ 1 MHz^20^,^21^.

In this work, we show that optomechanical coupling rates can be significantly enhanced by using photonic modes with subwavelength confinement. We realize a sliced photonic crystal nanobeam in which light is highly confined in a nanoscale volume near the moving dielectric interfaces of a low-mass resonator, leading to unprecedented interaction strengths. We use a simple free-space optical setup to address the structure and demonstrate optical tuning of the mechanical resonance frequency, as well as sensitive readout of mechanical motion. The observed optical forces and measurement sensitivity provide us with two independent ways to determine the vacuum coupling rate to be *g*_0_/2*π* ≈ 11.5 MHz. We demonstrate displacement readout with a detection imprecision below that at the standard quantum limit, i.e. with a noise level that is comparable to the quantum fluctuations of the resonator. We achieve this using only 22 μW of detected power even in a system with modest optical and mechanical quality factors. The operation with a relatively large cavity bandwidth is especially attractive for system integration and miniature sensor technologies as well as measurement-based control in nano-optomechanical systems. Simulations predict *g*_0_/2*π* can reach values of over 50 MHz with improvements in fabrication. This approach thus makes a significant step towards reaching the elusive regime of ultrastrong coupling (*g*_0_ > *κ*, Ω_m_), where nonclassical effects, such as the observation of quantum jumps of phonon number or the occurrence of photon blockade, arise directly from the optomechanical interaction even in the absence of strong driving^1^,^22^,^23^.

## Results

### Working principle

A displacement-induced frequency shift of an optical mode depends on the fraction of the energy density that is located near the moving dielectric boundaries^24^. Therefore, to realize a large photon-phonon coupling rate *g*_0_ = *Gx*_zpf_, the optical cavity mode should be localized at the positions where it is most influenced by the motion of the mechanical resonator. Crucially, it is most important to optimize the optical confinement along the directions in which the mechanical mode is also strongly localized.

**Figure 1:**
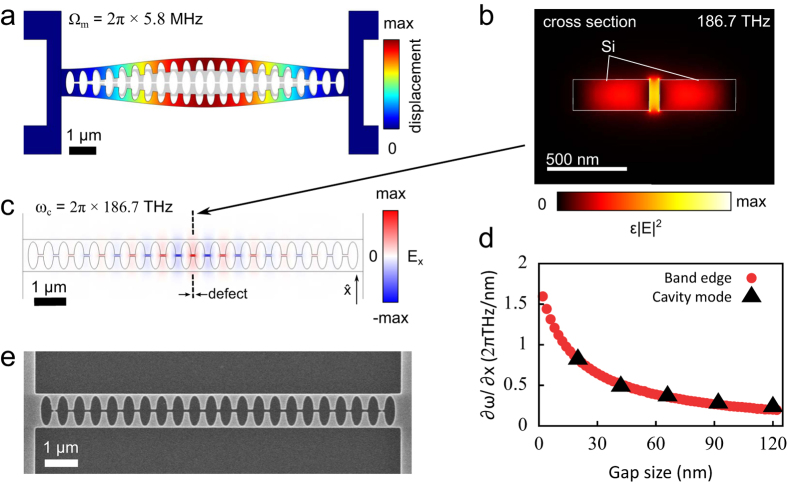
Resonances and geometry of the sliced nanobeam. (**a**) Simulated displacement profile of the fundamental (in-plane) mechanical resonance of the structure. (**b**) Cross section in the center of the sliced nanobeam (indicated by the dashed line in c), showing the simulated energy density distribution of the fundamental optical cavity mode of the structure. (**c**) Simulated transverse electric field profile of the fundamental optical cavity mode of the structure. (**d**) Simulated frequency shift as a result of an outward displacement of 1 nm. The cavity mode shift was determined by simulating the full nanobeam and introducing a uniform displacement along the beam. (**e**) Electron micrograph of a fabricated device. The thickness of the structure is 200 nm, both in the simulations and in the fabricated device.

The system we develop is based on a silicon photonic crystal nanobeam, which combines optical confinement with flexural mechanical motion (Fig. 1). The beam is ‘sliced’ through the middle such that it mechanically resembles a pair of doubly clamped beams, coupled through the clamping points at the ends of the nanobeam. We show the fundamental in-plane mechanical resonance of the sliced nanobeam in Fig. 1a. The small width (80 nm) of the narrowest parts of the half-beams ensures both the mass (≈ 2.4 pg) and the spring constant of the nanobeam are small, leading to large zero-point fluctuations *x*_zpf_.

The motion of the nanobeam effectively changes the local optical properties. The changes are strongly localized at the silicon surface perpendicular to the motion, but extend over several micrometers along the beam. The coupling rate will be largest when light is concentrated in the subwavelength gap separating the two halves. For this, we rely on the high localization of energy that can occur in systems with dielectric discontinuities with subwavelength dimensions^25^, in this case provided by the narrow gap itself.

To enable confinement along the length of the beam, we introduce a periodic patterning. This creates a photonic crystal, with a quasi-bandgap for transverse electric (TE) polarized modes guided by the beam (see Supplementary Information). The waveguide mode at the lower edge of the bandgap is strongly confined in the nanoscale gap separating the two half-beams (Fig. 1b). We introduce a defect in the photonic crystal by reducing the width of the central pair of ‘teeth’, such that the effective refractive index is locally reduced. This creates confined cavity modes with a frequency in the bandgap that are derived from the desired waveguide mode (Fig. 1c). Recently it was shown that with a similar approach photonic crystal nanobeam cavities can be created that simultaneously have a high quality factor and an ultrasmall mode volume^26^,^27^.

Like the mechanical mode, the field profile of the lowest-order optical cavity mode extends along the beam, but importantly is highly confined perpendicular to the motion (Fig. 1b,c). The truly subwavelength character of the transverse confinement is revealed by calculating the effective *mode area* of the waveguide mode it is derived from. We define the mode area as 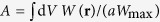, where the energy density *W*(**r**) = *ε*(**r**)|**E**(**r**)|^2^ has its maximum *W*_max_ just at the vacuum side of the gap boundary, and we integrate over a full unit cell with period *a*. The mode area is only 2.38 × 10^−14^ m^2^ for a gap width of 60 nm, or in other words *A* = 0.011*λ*^2^, with *λ* the wavelength in vacuum. In fact, it is even 8 times smaller than the squared wavelength *in silicon*, even though the maximum energy density is actually localized in the vacuum gap (Fig. 1b). This subwavelength mode area is essential to the sliced nanobeam and makes it stand out with respect to other designs, including the related double-beam ‘zipper’ cavity^8^,^15^,^18^,^28^, where the optical cavity modes of two photonic-crystal nanobeams are coupled by placing the beams close together.

Numerical simulations confirm that the frequency of both the band edge and the defect cavity mode derived from it respond strongly to a displacement of the two half-beams, reaching *G* = ∂*ω*_c_/∂*x* ≈ 2*π* × 0.4 THz/nm for a gap width of 60 nm (Fig. 1d). As expected, this value increases for smaller gap sizes *d*, due to an increase of the fraction of the energy in the gap^24^,^25^. We define the displacement coordinate as *x* = *d*/2 such that it can be directly related to the maximum lab-frame displacement of the antisymmetric mechanical mode depicted in Fig. 1a. Note that the choice of the definition of *x* is in principle arbitrary (with a properly matched definition of *m*_eff_), whereas the coupling rate *g*_0_ is independent of this definition. To determine the optical frequency shift, the entire half-beams are displaced in the simulation. The displacement of the actual mechanical mode is not uniform along the beam (Fig. 1a), meaning that due to the finite extent of the optical mode the value of *G* will be slightly reduced. Taking into account the optical and mechanical mode profiles (Fig. 1a,c), we estimate it to be 0.90 times the value shown in Fig. 1d (see Supplementary Information).

Using standard lithography techniques (see Methods), we realize free-standing sliced nanobeams in silicon with a thickness of 200 nm and a length of 11 μm, separated by an average gap size of 60 nm. An electron micrograph of a fabricated device is shown in Fig. 1e.

### Free-space readout

**Figure 2:**
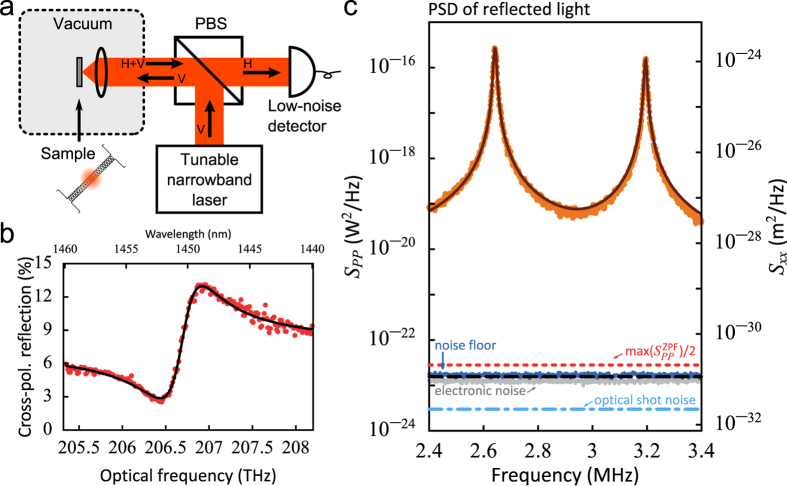
(**a**) Schematic diagram of the free-space readout method (PBS: polarizing beamsplitter; H,V: horizontally and vertically polarized light). See Methods for details. (**b**) Reflection spectrum (red datapoints) and fit with a Fano lineshape (black line). (**c**) Power spectral density of the reflected light obtained with the laser frequency on-resonance with the cavity (orange datapoints), and a fit of the two mechanical resonances (brown line). The noise floor (blue datapoints) was obtained by reflecting the laser light from the unpatterned substrate and matching the intensity on the detector. The black dashed line is the sum of the measured electronic noise (grey datapoints) and the optical shot noise calculated from the intensity on the detector (light blue dash-dotted line). The red dotted line shows the peak value of 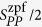 for the lowest-frequency resonance, which we obtained from the fit of the measured thermal spectrum via the relation 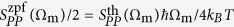.

We address our structure using a simple reflection measurement, schematically shown in Fig. 2a. The employed resonant scattering technique^29^ places the sample between crossed polarizers to allow the detection of light scattered by the cavity mode (whose dominant polarization is oriented at 45° to the polarizers) while suppressing light reflected by the substrate to significantly below 1%. By scanning the frequency of a narrowband laser we record the reflection spectrum, depicted for one of the samples in Fig. 2b. The dispersive lineshape is caused by interference of the resonant scattering of the cavity with non-resonant scattering by the nanobeam. The cross-polarized reflectance *R* is thus well fitted by a Fano lineshape^30^,^31^:


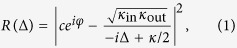


where *c* and *φ* are the amplitude and phase of the non-resonant scattering, respectively, and Δ ≡ *ω* − *ω*_c_ is the detuning of the laser frequency *ω* from the cavity resonance with linewidth *κ*. The rate at which light can couple to the cavity mode from the free-space input beam is given by *κ*_in_, whereas *κ*_out_ is the rate at which the cavity decays to the radiation channels that are detected through the output analyzer. In principle, these coupling rates can be unequal because the light emitted by the cavity has a spatial mode profile that differs from the Gaussian input beam.

Fitting equation (1) to the reflection spectrum yields the center frequency and linewidth of the cavity, as well as a value for 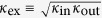. We determine *κ*_ex_ ≈ 0.29*κ* and the optical quality factor *Q*_*opt*_ = *ω*_c_/*κ* ≈ 400. Based on the large measured coupling efficiency, for these structures a non-cross-polarized measurement would likely have yielded good results as well (see Supplementary Information). The measured *Q*_opt_ is 2–3 times lower than the simulated one, a discrepancy that we attribute to fabrication imperfections.

Thermal motion of the nanobeam *δx* modulates the cavity frequency by *δω*_c_ = (∂*ω*_c_/∂*x*)*δx*. This produces a change in detected power proportional to the derivative of the reflection spectrum: *δP* = *P*_in_(∂*R*/∂*ω*_c_)*δω*_c_. Here we assumed the intracavity amplitude is instantaneously affected by the mechanical motion, which is justified since Ω_m_ ≪ *κ* (see Supplementary Information for the more general case). Thus, the power spectral densities of *x* and *P* are related as


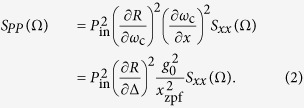


Figure 2c shows the detected spectral density *S*_*PP*_ for the laser tuned to the optical resonance frequency, with a relatively high optical power incident on the sample (*P*_in_ = 367 μW), corresponding to a detected power of 22 μW. Because of the linear relation between *S*_*PP*_ and *S*_*xx*_ shown in equation (2), this is a direct measurement of the spectrum of thermal motion in the nanobeam.

The two peaks at 2.6 and 3.2 MHz correspond to the two fundamental in-plane modes of the coupled halves of the nanobeam. For a perfectly symmetric structure, the (weak) coupling leads to two eigenmodes: a common mode, for which the half-beams move in phase and their separation *d* is not affected, and a differential mode, for which anti-phase movement of the half-beams results in maximal variation of *d*. Fabrication-related imperfections can break the symmetry of the system, such that the actual normal modes 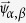 are linear combinations of the half-beam eigenmodes 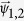
32: 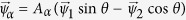 and 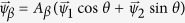, where *θ* can in principle take any value. As we show in the Supplementary Information, the frequencies of these mixed modes 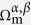 are reduced with respect to the simulated value of 6 MHz due to the presence of compressive stress in the studied sample, which also enhances the splitting between the two mode frequencies. Since the two modes generally affect the separation *d* differently, they have different photon-phonon coupling rates *g*_0_, which are maximal for a purely differential mode (*θ* = *π*/4). With our definition *x* = *d*/2, this is reflected in the fact that the ratio between the zero-point fluctuation amplitudes of the normal modes is 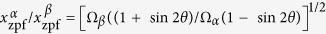 (see Supplementary Information). The variance in *x* due to thermal motion in the two modes is set by the equipartition theorem, taking into account this difference in *x*_zpf_. The ratio between the areas of the two resonance peaks in the experimental spectrum of *S*_*PP*_ therefore directly yields the mixing angle *θ*.

In fact, fitting two resonant modes to the displacement spectrum also allows determining the transduction factor that relates the measured optical power spectral density *S*_*PP*_ to the displacement spectrum *S*_*xx*_. To do so, we calculate the thermal variance 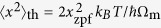. We determine *x*_zpf_ from the measured *θ* and from the effective mass of purely antisymmetric motion, which we computed from the simulated displacement profile to be *m*_eff_ ≈ 0.39*m*, with *m* the total mass of the beam. We further assume that the temperature *T* of the mechanical bath is equal to the lab temperature. The validity of this assumption is tested by performing power- and detuning-dependent measurements presented in the Supplementary Information. The resulting scale for the displacement spectral density *S*_*xx*_ is shown on the right side of Fig. 2c. Note that the chosen convention of *x* allows directly comparing the readout of the two mechanical resonances on this scale.

To determine the sensitivity with which the displacement spectrum of the beam can be read out, we consider the detection noise floor for the measurement shown in Fig. 2c, which is composed of electronic noise of the photodetector and the optical shot noise of the detected light. Their measured combined imprecision (blue datapoints in Fig. 2c) is over 7 orders of magnitude smaller than the measured signal.

A general assessment of the sensitivity capabilities of the measurement is made by comparing the detection noise imprecision to the (shot noise) imprecision 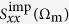 of a resonator read out at the standard quantum limit (SQL)^33^,^34^. The imprecision at the SQL is equal to half of the spectral density of the zero-point fluctuations 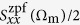. We determine this value from the measured thermal noise spectrum of the lowest-frequency mode via the average phonon occupancy of the mechanical mode *k*_*B*_*T*/*ħ*Ω_m_, and indicate it in Fig. 2c with the red dotted line. The optical shot noise of the light impinging on the detector, and even the total measurement noise floor, are lower than the imprecision noise at the SQL.

Readout of a nanomechanical resonator with an imprecision below that at the SQL was first achieved in 2009^33^,^34^ making use of high-quality optical and mechanical modes. These high quality factors were instrumental because the ability to perform a measurement with SQL-level sensitivity scales, per intracavity photon, with the single-photon cooperativity 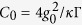. This shows it depends on the photon-phonon coupling strength as well as the optical linewidth *κ* = *ω*_c_/*Q*_opt_ and the mechanical linewidth Γ = Ω_m_/*Q*_m_. The fact that here we achieve a detection noise imprecision below that at the SQL with optical and mechanical quality factors of both less than 500 attests to the large optomechanical coupling strength, and could have important application in broadband, sensitive nanoscale sensors.

### Determining the photon-phonon coupling rate

To quantify the optomechanical interaction strength in the fabricated devices, we model the transduction of thermal displacement fluctuations using equation (2) and use it to fit a low-power measurement on a structure for various laser detunings. We do this by calculating the variance of the optical power fluctuations *δP* at the detector resulting from displacement fluctuations *δx* of a mechanical mode with known (thermal) variance. Integrating equation (2) over a single mechanical mode and using our expression for the reflection spectrum *R*(Δ) (equation (1)), yields


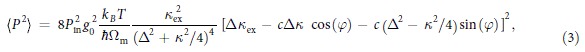


which is independent of the choice of the displacement coordinate *x*.

**Figure 3:**
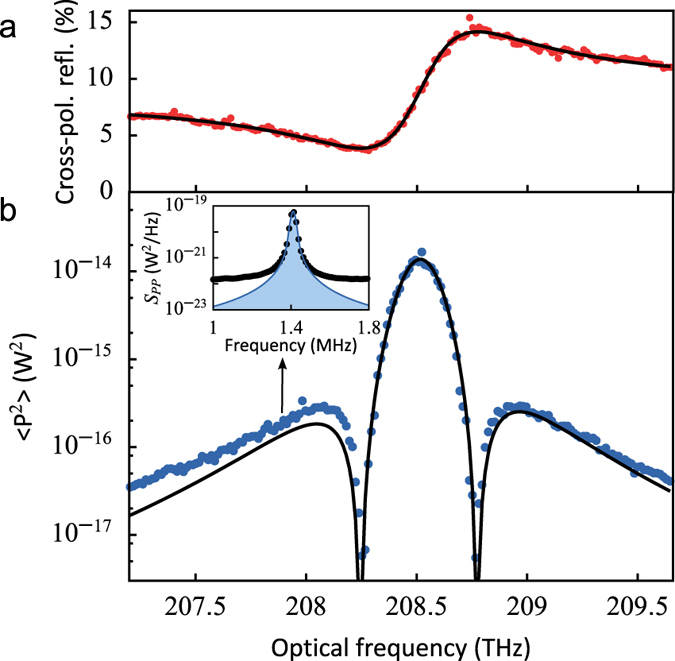
Sensitivity of the displacement measurement. (**a**) Reflection spectrum (red datapoints) and fit with Fano lineshape (black line) for this particular nanobeam. (**b**) Detected optical variance in the fundamental mechanical resonance (blue datapoints) with power incident on the sample *P*_in_ = 8.5 μW. The datapoints were obtained by fitting the fundamental mechanical resonance peak in the measured modulation spectra (inset). The signal originates from thermal motion so it varies only with the sensitivity of the measurement. The black line shows our model, which uses the parameters obtained from the reflection spectrum in (**a**) and is fitted to the data to determine *g*_0_/2*π* = 11.5 MHz.

The measured variance of the optically modulated signal due to the lowest-frequency mechanical mode is shown in Fig. 3b. The variance is minimal when the derivative of the reflection signal (Fig. 3a) vanishes. Interestingly, due to the dispersive lineshape the transduction is largest for the laser tuned to resonance. The line shown in Fig. 3b is a fit of equation (3) to the data, using only *g*_0_ as a free fitting parameter (all other parameters having been determined in independent measurements). The corresponding value for *g*_0_/2*π* is 11.5 MHz, which is an order of magnitude larger than previously reported values^4^,^8^,^15^,^16^,^19^,^20^,^21^.

To compare this photon-phonon coupling rate to the prediction from our simulation we estimate the zero-point fluctuations of the structure. Using the measured mechanical resonance frequency and the simulated effective mass, we obtain 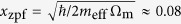 pm for a purely anti-symmetric mode. With the simulated frequency response *G*, this yields a prediction of *g*_0_/2*π* ≈ 26 MHz. To take into account the observed asymmetry of the mechanical mode, we should apply a correction factor of 0.76, based on our knowledge of *θ* (see Supplementary Information). This results in an expected value of *g*_0_/2*π* ≈ 20 MHz. We attribute the remaining discrepancy to fabrication imperfections, which could result in a different overlap of the optical and mechanical modes than simulated. This implies that a further increase of *g*_0_ even beyond the measured value is possible. In fact, we expect to be able to improve the fabrication process to produce smaller gaps, which increases the coupling rate significantly (Fig. 1d). If we for example assume a gap width of 25 nm and an antisymmetric mode, the simulations predict *g*_0_/2*π* reaches a value as large as 53 MHz.

### Optical spring tuning

**Figure 4:**
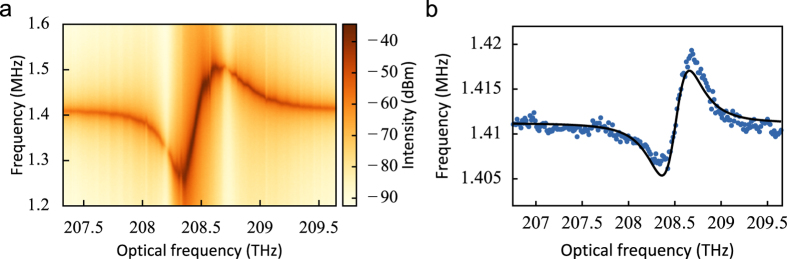
(**a**) Spectrogram showing optical tuning of mechanical resonance frequency with power incident on the sample *P*_in_ = 140 μW. (**b**) Fitted frequency of the fundamental mechanical resonance (blue datapoints) with *P*_in_ = 8.5 μW. The black line shows a fit using the model for optical spring tuning (equation (4)).

While we tune the laser frequency across the optical resonance a pronounced shift of the mechanical resonance frequency is observed. In Fig. 4a this is shown for the same structure we studied in Fig. 3. This well-known optical spring effect is caused by the radiation pressure force being opposed to (aligned with) the mechanical restoring force when the laser is detuned below (above) the resonance frequency, changing the effective spring constant and therefore the mechanical resonance frequency^1^. The equation that describes this behaviour in the limit of a large cavity linewidth (*κ* ≫ Ω_m_) is


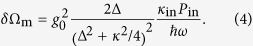


From equation (4) we recognize that the optical spring tuning shown in Fig. 4 provides a second, independent way to characterize the photon-phonon coupling rate. Figure 4b shows the center frequency of the mechanical resonance extracted from the same measurement as the variances in Fig. 3b, as well as a fit using equation (4). To estimate *g*_0_ from this fit we need to know *κ*_in_, which we cannot easily determine as it generally depends on the overlap between the focused Gaussian beam and the cavity mode profile. However, we can find bounds for *κ*_in_ by considering the total decay rate *κ* and 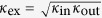, which were determined from the fit to the reflection spectrum. On the one hand we know *κ*_in_ ≤ *κ*_ex_, i.e. the collection efficiency is at least as efficient as the overlap with a Gaussian beam, and on the other hand 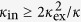, i.e. at most half of the light escaping from the cavity can be collected because of the vertical symmetry of the structure. Combining these bounds with the fit of the optical spring effect yields a range for *g*_0_ between 10 and 13 MHz. This range is in good agreement with the value obtained from the analysis of measurement transduction, and moreover both are consistent with the theoretical predictions. Therefore no alternate transduction mechanisms need to be invoked to explain the results. Additionally, the fact that the spring shift can be fully explained by the radiation pressure force as predicted by equation (4) shows that forces due to photothermoelastic effects^35^ are likely insignificant compared to radiation pressure.

### Nonlinear transduction

As a consequence of the large photon-phonon coupling rate, the thermal motion of the nanobeam (*δx*_rms_ ≈ 230 pm) induces frequency changes 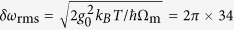 GHz, which is appreciable with respect to the linewidth of the cavity. The resulting nonlinear transduction generates spurious signals at integer multiples of, and combinations of, the strongest modulation frequencies. Detection of such signals at multiples of the mechanical resonance frequency resulting from thermal motion was reported previously^18^,^36^,^37^ and compared to quadratic optomechanical coupling^38^.

**Figure 5:**
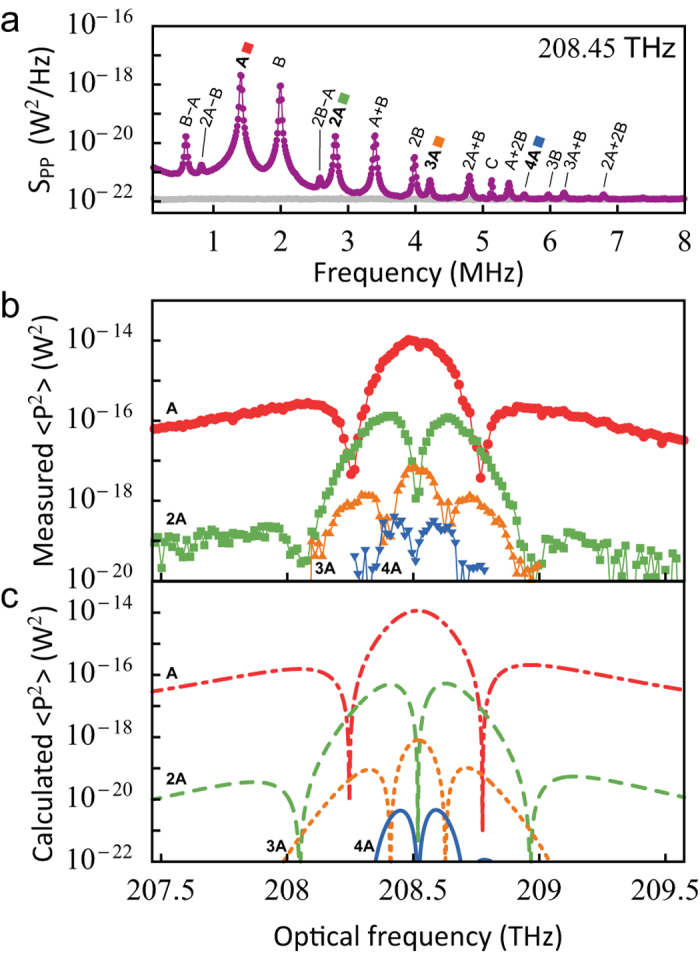
Nonlinear transduction. (**a**) Measured mechanical spectrum of the sliced nanobeam (purple datapoints), using near-resonant light with power incident on the sample *P*_in_ = 8.5 μW. Three peaks A, B and C are mechanical resonances of the nanobeam, all other visible peaks correspond to integer multiples and combinations of frequencies A and B. The electronic noise floor is shown with grey datapoints. (**b**) Areas under the peaks corresponding to integer multiples of frequency A, obtained by fitting the peaks in the spectrum. (**c**) Calculated variance of the reflected signal, using the experimentally obtained parameters.

Figure 5a shows a transduced spectrum where we identify 15 peaks as integer multiples and combinations of the two fundamental mechanical resonances at 1.4 MHz (“A”) and 2.0 MHz (“B”): Ω_*j*,*k*_ = |*jA* ± *k*B|, with *j*, *k* ∈ {0, 1, 2,…}. Peaks corresponding to different order (*j* + *k*) have a different detuning dependence, but all peaks with the same order differ only by a constant factor. This further confirms our identifying them as nonlinear transduction peaks instead of separate mechanical resonances. To illustrate the detuning dependence of the higher-order peaks, we plot the variance of the peaks *j*A for *j* = {1, 2, 3, 4} in Fig. 5b.

The detected height of the higher-order peaks can be predicted by a Taylor expansion of the amount of light in the cavity around the average detuning^38^ (see Supplementary Information), the result of which is shown in Fig. 5c. Note that the higher-order peaks in this calculation were not fitted to the data, but follow from the value of *g*_0_ we obtained by fitting the first-order peak, as shown in Fig. 3. The measured nonlinear sidebands are larger than expected (corresponding to a suggested increase of *g*_0_ of about 60%). The origin of this discrepancy is unknown. Possible explanations include higher-order optomechanical coupling^38^ or mechanical nonlinearities^39^. However, the symmetry and shape of the curves match the experimental data, which confirms that the detuning dependence corresponds to the successive derivatives of the reflection spectrum (Fig. 3a).

## Discussion

The free-space readout method we employ provides an easy and robust way of coupling light to the cavity. We have intentionally engineered the cavity defect such that it has a significant dipole moment^40^, allowing coupling to free space at an appreciable rate. This makes it unnecessary to create an explicit loss channel for coupling, e.g. in the form of a grating or feeding waveguide. The currently achieved coupling rate of *κ*_ex_ = 0.29*κ* is comparable to that achieved with standard tapered fibre coupling (e.g. *κ*_ex_ = 0.13*κ* by Chan *et al.*^4^). In both cases, it is possible to engineer the system such that it couples more efficiently, as has recently been shown for fibre coupling^37^. The Fano-shape of the reflection spectrum allows direct transduction of motion to optical amplitude modulation for a laser tuned to the cavity resonance (where dynamical radiation pressure backaction is zero), without more complicated interferometric schemes. As a result of the efficient coupling to free space, the bandwidth of the cavity is large (0.5 THz), which is appealing in the context of applications that require frequency matching of multiple systems: together with the small system footprint, it could assist the integration of such optomechanical transducers in sensor arrays^41^ or effective optomechanical metamaterials^42^.

Of course, for applications that benefit from enhanced measurement sensitivity such as measurement-based control of the mechanical quantum state, it could be worthwhile to realize a higher optical quality factor by introducing tapering along the nanobeam^26^,^40^. To simultaneously allow efficient free-space coupling would in such a case require special attention, in the form of tailoring the spatial mode profile of the cavity radiation. This could be especially important for effects that depend on the intracavity photon number, such as the demonstrated optical spring effect, as the rates *κ*_in_ and *κ*_out_ will differ. Further quantification of their individual magnitudes (e.g. through systematic variation of incident and detected mode profiles) will thus be valuable.

Likewise, we expect that the mechanical quality factor for the nanobeams we employ can be improved with suitable design principles and optimization of the fabrication process. Indeed, measurements on similar-sized silicon nanobeams and cantilevers suggest that quality factors in the range of 10^4^ to 10^5^ should be possible at room and cryogenic temperature, respectively^8^,^43^. Nonetheless, we point out that because of the large coupling rate, even with the current modest values of both optical and mechanical quality factors the single-photon cooperativity in this structure reaches *C*_0_ = 0.16. The value of this quantity, which compares optomechanical coupling strength and dissipation, and is for example a measure for the capability of the system to perform measurements at the SQL, is on par with many recently reported systems with much higher quality factors^1^.

In conclusion, we demonstrated an optomechanical device with a large photon-phonon coupling rate *g*_0_/2*π* = 11.5 MHz, and used it to demonstrate sensitive measurement of nanomechanical motion and pronounced optical tuning of the mechanical resonance frequency. It is interesting to note that the regime of large coupling rate and modest optical linewidth is beneficial in the context of achieving strong mechanical tuning, as parametric instability is suppressed. We revealed that the working mechanism relies on an optical mode with a subwavelength mode area. We predict this approach can be extended to yield even larger coupling rates, or to be applied to modes with higher mechanical frequencies. In the current device the photon-phonon coupling rate *g*_0_ exceeds the mechanical resonance frequency Ω_m_, which is one of the requirements for ultrastrong coupling^1^,^22^,^23^. With further improvements in both the coupling rate and the optical quality factor, the present approach might provide a route to simultaneously reach *g*_0_ > Ω_m_ and *g*_0_ ≈ *κ*. It will be interesting to explore to what extent this regime can be used to exploit nonlinear optomechanical interactions at the single-photon level.

## Methods

### Numerical simulation

All numerical eigenmode simulations were performed using finite-element software COMSOL Multiphysics. In mechanical simulations, the connection between the substrate and the support pads was modeled as a fixed boundary, while all other boundaries were kept free. To find the guided modes of the photonic crystal nanobeam, a unit cell was simulated with Floquet boundary conditions along the propagation direction and in the other directions perfect electric conductors at several micrometers distance from the structure. Finally, to simulate the cavity mode, a full nanobeam including support pad was modeled with perfectly matched layers on all sides, again at several micrometers distance from the beam.

### Fabrication

The structures were fabricated from a silicon-on-insulator wafer with a device layer thickness of 200 nm, and a buried oxide layer of 1 μm thick. A resist layer of hydrogen silsesquioxane (HSQ) with a thickness of 80 nm was spincoated on top and patterned using electrons accelerated with 30 kV. The resist was developed using TMAH and then the pattern was transferred to the silicon layer using a reactive-ion etch process with SF_6_/O_2_ gases, optimized for anisotropy and selectivity. To release the structures, the oxide layer was dissolved in a 20% HF solution. After this step the structure was dried with a critical point dryer to prevent the sliced beams being pulled together during the drying process. The suspension of the nanobeams from their support pads was designed to allow some relief of compressive stress along the beam. The compressive stress is present in most free-standing structures created from SOI^44^, but it has a large effect for our structures because of their low stiffness.

### Free-space setup

The laser beam (New Focus Velocity 6725) was focused on the sample by an aspheric lens with a numerical aperture of 0.6. We estimate the resulting spot size to be near the diffraction-limited value of 2.8 μm. A polarizing beamsplitter provided a cross-polarized detection scheme, where any light that was directly reflected was rejected and only light that coupled to the sample, placed at 45°, was transmitted to the detector. Both the lens and the sample were in a vacuum chamber to reduce mechanical damping by air molecules. The spectra in Fig. 2 were taken while intermittently running the vacuum pump to obtain a pressure lower than 10^−3^ mbar. All other experimental results shown were obtained with the vacuum pump turned off, at a higher pressure of about 4 mbar. While the higher pressure lowered the mechanical quality factor to approximately 200, the subsequent analysis was independent of the mechanical linewidth.

### Analysis of modulated reflection signals

We detected the reflection signal using a low-noise InGaAs-based photoreceiver (Femto HCA-S) and analyzed it using an electronic spectrum analyzer (Agilent MXA). We fitted the peaks in the modulation spectra using a Lorentzian convolved with a Gaussian distribution, also called a Voigt lineshape. The Gaussian contribution accounted for the resolution bandwidth of the spectrum analyser, as well as for frequency-noise broadening at relatively large optical input power *P*_in_. With high *P*_in_, small fluctuations in incoupling efficiency or laser intensity thermally shifted the cavity resonance, which resulted in frequency noise via optical spring tuning of the mechanical resonance.

## Additional Information

**How to cite this article**: Leijssen, R. and Verhagen, E. Strong optomechanical interactions in a sliced photonic crystal nanobeam. *Sci. Rep.* **5**, 15974; doi: 10.1038/srep15974 (2015).

## Supplementary Material

Supplementary Information

## References

[b1] Aspelmeyer M., Kippenberg T. J. & Marquardt F. Cavity optomechanics. Rev. Mod. Phys. 86, 1391–1452 (2014).

[b2] Vanner M. R., Pikovski I. & Kim M. S. Towards optomechanical quantum state reconstruction of mechanical motion. Ann. Phys. 527, 15–26 (2015).

[b3] Teufel J. D. *et al.* Sideband cooling of micromechanical motion to the quantum ground state. Nature 475, 359–363 (2011). 21734657 10.1038/nature10261

[b4] Chan J. *et al.* Laser cooling of a nanomechanical oscillator into its quantum ground state. Nature 478, 89–92 (2011). 21979049 10.1038/nature10461

[b5] Verhagen E., Deléglise S., Weis S., Schliesser A. & Kippenberg T. J. Quantum-coherent coupling of a mechanical oscillator to an optical cavity mode. Nature 482, 63–67 (2012). 22297970 10.1038/nature10787

[b6] Cohadon P. F., Heidmann A. & Pinard M. Cooling of a Mirror by Radiation Pressure. Phys. Rev. Lett. 83, 3174–3177 (1999).

[b7] Wilson D. J. *et al.* Measurement-based control of a mechanical oscillator at its thermal decoherence rate. Nature 524, 325–329 (2015). 26258303 10.1038/nature14672

[b8] Safavi-Naeini A. H. *et al.* Squeezed light from a silicon micromechanical resonator. Nature 500, 185–189 (2013). 23925241 10.1038/nature12307

[b9] Purdy T. P., Peterson R. W. & Regal C. A. Observation of radiation pressure shot noise on a macroscopic object. Science 339, 801–804 (2013). 23413350 10.1126/science.1231282

[b10] Vanner M. R., Hofer J., Cole G. D. & Aspelmeyer M. Cooling-by-measurement and mechanical state tomography via pulsed optomechanics. Nat. Commun. 4, 2295 (2013). 23945768 10.1038/ncomms3295

[b11] Gavartin E., Verlot P. & Kippenberg T. J. A hybrid on-chip optomechanical transducer for ultrasensitive force measurements. Nat. Nanotechnol. 7, 509–514 (2012). 22728341 10.1038/nnano.2012.97

[b12] Poggio M., Degen C. L., Mamin H. J. & Rugar D. Feedback Cooling of a Cantilevers Fundamental Mode below 5 mK. Phys. Rev. Lett. 99, 017201 (2007). 17678185 10.1103/PhysRevLett.99.017201

[b13] Vanner M. R. *et al.* Pulsed quantum optomechanics. Proc. Natl. Acad. Sci. USA. 108, 16182–16187 (2011). 21900608 10.1073/pnas.1105098108 PMC3182722

[b14] Genes C., Vitali D., Tombesi P., Gigan S. & Aspelmeyer M. Ground-state cooling of a micromechanical oscillator: Comparing cold damping and cavity-assisted cooling schemes. Phys. Rev. A 77, 033804 (2008).

[b15] Eichenfield M., Camacho R., Chan J., Vahala K. J. & Painter O. A picogram- and nanometre-scale photonic-crystal optomechanical cavity. Nature 459, 550–555 (2009). 19489118 10.1038/nature08061

[b16] Gomis-Bresco J. *et al.* A one-dimensional optomechanical crystal with a complete phononic band gap. Nat. Commun. 5, 4452 (2014). 25043827 10.1038/ncomms5452

[b17] Gavartin E. *et al.* Optomechanical Coupling in a Two-Dimensional Photonic Crystal Defect Cavity. Phys. Rev. Lett. 106, 203902 (2011). 21668229 10.1103/PhysRevLett.106.203902

[b18] Deotare P. B. *et al.* All optical reconfiguration of optomechanical filters. Nat. Commun. 3, 846 (2012). 22617286 10.1038/ncomms1830

[b19] Ding L. *et al.* Wavelength-sized GaAs optomechanical resonators with gigahertz frequency. Appl. Phys. Lett. 98, 113108 (2011).

[b20] Balram K. C., Davanço M., Lim J. Y., Song J. D. & Srinivasan K. Moving boundary and photoelastic coupling in GaAs optomechanical resonators. Optica 1, 414–420 (2014).

[b21] Chan J., Safavi-Naeini A. H., Hill J. T., Meenehan S. & Painter O. Optimized optomechanical crystal cavity with acoustic radiation shield. Appl. Phys. Lett. 101, 081115 (2012).

[b22] Nunnenkamp A., Børkje K. & Girvin S. M. Single-Photon Optomechanics. Phys. Rev. Lett. 107, 063602 (2011). 21902323 10.1103/PhysRevLett.107.063602

[b23] Yeo I. *et al.* Strain-mediated coupling in a quantum dot-mechanical oscillator hybrid system. Nat. Nanotechnol. 9, 106–110 (2014). 24362234 10.1038/nnano.2013.274

[b24] Johnson S. *et al.* Perturbation theory for Maxwells equations with shifting material boundaries. Phys. Rev. E 65, 066611 (2002). 10.1103/PhysRevE.65.066611 12188855

[b25] Robinson J. T., Manolatou C., Chen L. & Lipson M. Ultrasmall Mode Volumes in Dielectric Optical Microcavities. Phys. Rev. Lett. 95, 143901 (2005). 16241653 10.1103/PhysRevLett.95.143901

[b26] Ryckman J. D. & Weiss S. M. Low mode volume slotted photonic crystal single nanobeam cavity. Appl. Phys. Lett. 101, 071104 (2012).

[b27] Seidler P., Lister K., Drechsler U., Hofrichter J. & Stoferle T. Slotted photonic crystal nanobeam cavity with an ultrahigh quality factor-to-mode volume ratio. Opt. Express 21, 32468–32483 (2013). 24514840 10.1364/OE.21.032468

[b28] Gong Y., Rundquist A., Majumdar A. & Vučković J. Low power resonant optical excitation of an optomechanical cavity. Opt. Express 19, 1429–1440 (2011). 21263684 10.1364/OE.19.001429

[b29] McCutcheon M. W. *et al.* Resonant scattering and second-harmonic spectroscopy of planar photonic crystal microcavities. Appl. Phys. Lett. 87, 221110 (2005).

[b30] Fan S., Suh W. & Joannopoulos J. D. Temporal coupled-mode theory for the Fano resonance in optical resonators. J. Opt. Soc. Am. A 20, 569 (2003). 10.1364/josaa.20.000569 12630843

[b31] Galli M. *et al.* Light scattering and Fano resonances in high-Q photonic crystal nanocavities. Appl. Phys. Lett. 94, 071101 (2009).

[b32] Sun X., Zheng J., Poot M., Wong C. W. & Tang H. X. Femtogram doubly clamped nanomechanical resonators embedded in a high-Q two-dimensional photonic crystal nanocavity. Nano Lett. 12, 2299–2305 (2012). 22471420 10.1021/nl300142t

[b33] Anetsberger G. *et al.* Near-field cavity optomechanics with nanomechanical oscillators. Nat. Phys. 5, 909–914 (2009).

[b34] Teufel J. D., Donner T., Castellanos-Beltran M. A., Harlow J. W. & Lehnert K. W. Nanomechanical motion measured with an imprecision below that at the standard quantum limit. Nat. Nanotechnol. 4, 820–823 (2009). 19893515 10.1038/nnano.2009.343

[b35] Metzger C., Favero I., Ortlieb A. & Karrai K. Optical self cooling of a deformable Fabry-Perot cavity in the classical limit. Phys. Rev. B 78, 035309 (2008).

[b36] Lin Q., Rosenberg J., Jiang X., Vahala K. J. & Painter O. Mechanical Oscillation and Cooling Actuated by the Optical Gradient Force. Phys. Rev. Lett. 103, 103601 (2009). 19792308 10.1103/PhysRevLett.103.103601

[b37] Cohen J. D., Meenehan S. M. & Painter O. Optical coupling to nanoscale optomechanical cavities for near quantum-limited motion transduction. Opt. Express 21, 11227–11236 (2013). 23669980 10.1364/OE.21.011227

[b38] Doolin C. *et al.* Nonlinear optomechanics in the stationary regime. Phys. Rev. A 89, 053838 (2014).

[b39] Ramos D., Frank I. W., Deotare P. B., Bulu I. & Lončar M. Non-linear mixing in coupled photonic crystal nanobeam cavities due to cross-coupling opto-mechanical mechanisms. Appl. Phys. Lett. 105, 181121 (2014).

[b40] Quan Q., Deotare P. B. & Lončar M. Photonic crystal nanobeam cavity strongly coupled to the feeding waveguide. Appl. Phys. Lett. 96, 203102 (2010).

[b41] Thijssen R., Kippenberg T. J., Polman A. & Verhagen E. Parallel Transduction of Nanomechanical Motion Using Plasmonic Resonators. ACS photonics 1, 1181–1188 (2014). 25642442 10.1021/ph500262b PMC4307941

[b42] Heinrich G., Ludwig M., Qian J., Kubala B. & Marquardt F. Collective Dynamics in Optomechanical Arrays. Phys. Rev. Lett. 107, 043603 (2011). 21867004 10.1103/PhysRevLett.107.043603

[b43] Tao Y., Boss J. M., Moores B. A. & Degen C. L. Single-crystal diamond nanomechanical resonators with quality factors exceeding one million. Nat. Commun. 5, 3638 (2014). 24710311 10.1038/ncomms4638

[b44] Yamashita D., Takahashi Y., Asano T. & Noda S. Raman shift and strain effect in high-Q photonic crystal silicon nanocavity. Opt. Express 23, 3951 (2015). 25836434 10.1364/OE.23.003951

