# Supplementary material for: Strong optomechanical interactions in a sliced photonic crystal nanobeam
Source: Sci Rep. 2015 Nov 2;5:15974. doi: 10.1038/srep15974 (PMC4629158; doi:10.1038/srep15974)
Supplement: Supplementary Information [file srep15974-s1.pdf]

Supplementary material
Strong optomechanical interactions in a sliced photonic crystal nanobeam

Rick Leijssen[1] **and Ewold Verhagen**[1,*]

[1]Center for Nanophotonics, FOM Institute AMOLF, Science Park 104, 1098 XG, Amsterdam, The Netherlands
[*]Corresponding author: verhagen@amolf.nl

ABSTRACT

This document provides supplementary information to "Strong optomechanical interactions in a sliced photonic crystal nanobeam". It contains extra information on the model used for analysis, numerical simulations showing properties of the device's resonant modes, and analysis of the influence of non-ideal fabrication, of high optical input power, and of the cross-polarized detection scheme.

Model for transduction

We derive the effect of a small frequency modulation of the intracavity field at the output of a cavity, which allows us to set up a model that describes the transduction of thermal motion to optical intensity modulation. This derivation follows the calculation shown by Gorodetsky *et al.*,[1] with the important difference that we include the non-resonant contribution in the reflection spectrum that leads to the Fano lineshape we observe.

We start from the equations describing the behaviour of the optomechanical system

$$\dot{a} = [i(\Delta - Gx(t)) - \kappa/2]a(t) + \sqrt{\kappa_{\text{in}}}s_{\text{in}}(t), \tag{S1}$$

$$\ddot{x}(t) + \Gamma_{\text{m}}\dot{x}(t) + \Omega_{\text{m}}^2 x(t) = -\hbar G|\bar{a}|^2, \tag{S2}$$

where a is the internal field in the cavity, s_{in} is the input field related to the input power $P_{\text{in}} = \hbar\omega|s_{\text{in}}|^2$, Δ is the detuning of the input light from the cavity resonance ω_{c}, κ is the cavity decay rate, κ_{in} is the coupling rate to the input channel and $G = \partial\omega_{\text{c}}/\partial x$ is the optomechanical frequency response. The frequency Ω_{m} and damping rate Γ_m of the mechanical resonator are influenced by the number of photons in the cavity $|\bar{a}|^2$, an effect we neglect in the following by assuming a low input power. This simplification is motivated by the fact that we seek to predict the amplitude of the mechanically-induced light modulation, not the frequency of such modulations. Moreover, dynamical backaction affecting mechanical linewidth is small in devices that have large κ/Ω_{m}.

We consider a small harmonic oscillation of the mechanical resonator $x(t) = x_0\cos(\Omega_{\text{m}}t)$, which causes a modulation of the cavity frequency with amplitude x_0G, or a modulation of the optical intracavity phase with amplitude x_0G/Ω_{m}. If the modulation is small ($x_0G \ll \kappa$), this yields

$$\begin{aligned} a_x &= s_{\text{in}}\sqrt{\kappa_{\text{in}}}\mathscr{L}(0) \\ &\quad\times\left[1 - \frac{i}{2}x_0G\mathscr{L}(\Omega_{\text{m}})e^{-i\Omega_{\text{m}}t} - \frac{i}{2}x_0G\mathscr{L}(-\Omega_{\text{m}})e^{i\Omega_{\text{m}}t}\right], \\ \mathscr{L}(\Omega) &= \frac{1}{-i(\Delta+\Omega)+\kappa/2}. \end{aligned} \tag{S3}$$

The cavity is coupled to the output s_{out} with the coupling rate κ_{out}. The equation for the output field reads

$$s_{\text{out}} = ce^{i\varphi}s_{\text{in}} - \sqrt{\kappa_{\text{out}}}a_x, \tag{S4}$$

where the first term is caused by the nonresonant scattering from the input to the output with amplitude c and phase φ.

In the experiment, we measure the intensity $|s_{\text{out}}|^2$ and feed it to a spectrum analyser, which yields the single-sided spectrum of the signal. Since we are interested in the strength of the spectral component at the mechanical oscillation frequency Ω_{m}, we highlight the time dependence here:

$$|s_{\text{out}}|^2(t) = c^2|s_{\text{in}}|^2 + \kappa_{\text{out}}|a_x(t)|^2 - c\sqrt{\kappa_{\text{out}}}[e^{i\varphi}s_{\text{in}}a_x^*(t) + e^{-i\varphi}s_{\text{in}}^*a_x(t)]. \tag{S5}$$

The first term is constant so it does not contribute to a signal at Ω_m. Substituting a_x into the other two terms, and discarding any terms not oscillating at $\pm\Omega_\mathrm{m}$ yields

$$|s_\mathrm{out}|^2(t)\Big|_{\pm\Omega_\mathrm{m}} = \frac{1}{2}x_0 G|s_{in}|^2\sqrt{\kappa_{in}\kappa_{out}} \times \Bigg[\sqrt{\kappa_{in}\kappa_{out}}|\mathscr{L}(0)|^2\left(\left\{ie^{i\Omega_\mathrm{m}t}\left[\mathscr{L}^*(\Omega_\mathrm{m})-\mathscr{L}(-\Omega_\mathrm{m})\right]\right\}+\text{c.c.}\right)$$
$$-c\left(\left\{ie^{i\Omega_\mathrm{m}t}\left[e^{i\varphi}\mathscr{L}^*(0)\mathscr{L}^*(\Omega_\mathrm{m})-e^{-i\varphi}\mathscr{L}(0)\mathscr{L}(-\Omega_\mathrm{m})\right]\right\}+\text{c.c.}\right)\Bigg]. \quad \text{(S6)}$$

This expression contains the modulation amplitude. In our experiment, we compare the variance of the modulation to the known variance of the mechanical thermal motion $\langle x^2\rangle_\mathrm{th} = 2x_\mathrm{zpf}^2 k_B T/\hbar\Omega_\mathrm{m}$. Therefore we calculate the variance of $P_\mathrm{out} = \hbar\omega_0|s_{out}|^2$ due to the modulation at $+\Omega_\mathrm{m}$ and $-\Omega_\mathrm{m}$, which will both contribute to the signal at $+\Omega_\mathrm{m}$ in the single-sided spectrum. We can write $P_\mathrm{out}\Big|_{\pm\Omega_\mathrm{m}} = Ae^{i\Omega_\mathrm{m}t}+A^*e^{-i\Omega_\mathrm{m}t}$, which leads to $\langle|P_\mathrm{out}|^2\rangle_{\Omega_\mathrm{m}} = 2|A|^2$. After some algebra, we arrive at

$$\langle|P_\mathrm{out}|^2\rangle_{\Omega_\mathrm{m}} = \frac{2x_0^2G^2P_{in}^2(\kappa_{in}\kappa_{out})}{(\Delta^2+\kappa^2/4)^2[(\Delta+\Omega_\mathrm{m})^2+\kappa^2/4][(\Delta-\Omega_\mathrm{m})^2+\kappa^2/4]}\Big\{\Delta^2\kappa_{in}\kappa_{out}-2\Delta c\sqrt{\kappa_{in}\kappa_{out}}[\Delta\kappa\cos\varphi+(\Delta^2-\kappa^2/4)\sin\varphi]$$
$$+c^2\left[\Delta^2(\Omega_\mathrm{m}^2+\kappa^2)\cos^2\varphi+(\Delta^4-\Delta^2\kappa^2/2+\kappa^2\Omega_\mathrm{m}^2/4+\kappa^4/16)\sin^2\varphi-2\cos\varphi\sin\varphi(-\Delta^3\kappa+\Delta\kappa^3/4+\Delta\kappa\Omega_\mathrm{m}^2/2)\right]\Big\}. \quad \text{(S7)}$$

If we evaluate this expression in the bad-cavity limit ($\Omega_\mathrm{m} \ll \kappa$), we find it is directly related to the derivative of the Fano lineshape $\partial R/\partial\Delta$:

$$\begin{aligned}\langle|P_\mathrm{out}|^2\rangle_{\Omega_\mathrm{m}} &= \frac{2x_0^2G^2P_\mathrm{in}^2\kappa_\mathrm{in}\kappa_\mathrm{out}\left[\Delta\sqrt{\kappa_\mathrm{in}\kappa_\mathrm{out}}-c\Delta\kappa\cos\varphi-c(\Delta^2-\kappa^2/4)\sin\varphi\right]^2}{(\Delta^2+\kappa^2/4)^4}\\ &= \frac{1}{2}x_0^2G^2P_\mathrm{in}^2\left(\frac{\partial R}{\partial\Delta}\right)^2.\end{aligned} \quad \text{(S8)}$$

We note that imperfect transmission of the optics between the sample and the detector scales the detected signal in the same way as the input power P_in, and will enter the equations in the same way. Finally, we substitute the variance due to the modulation amplitude x_0 by the variance of the thermal mechanical motion: $x_0^2G^2 \to 2\langle x^2\rangle_\mathrm{th}G^2 = 4g_0^2k_BT/\hbar\Omega_\mathrm{m}$, which leads to equation (3) in the main text.

Nonlinear transduction

The previous section started from the assumption that the frequency modulation $\delta\omega_\mathrm{c} = G\delta x$ is small with respect to the cavity linewidth, $\delta\omega_\mathrm{c} \ll \kappa$, and considered only the resulting linear transduction at the modulation frequency Ω_m. In this section we show that the first signature of large $\delta\omega_\mathrm{c}$ is the appearance of nonlinear transduction, which produces a signal at multiples of the modulation frequency Ω_m. For the second-order transduction, this was shown by Doolin *et al.*,[2] where also a quadratic optomechanical coupling was taken into account. Here we derive the result for any higher-order terms of nonlinear transduction.

In the non-resolved sideband regime ($\kappa \gg \Omega_\mathrm{m}$), the optical fields in the cavity reach a steady state much faster than the timescale of mechanical motion. The intracavity amplitude can then be written as

$$a(t) = \frac{\sqrt{\kappa_\mathrm{in}}s_\mathrm{in}}{-i[\Delta-\delta\omega_\mathrm{c}(t)]+\kappa/2}, \quad \text{(S9)}$$

which combined with equation (S4) yields

$$\frac{|s_\mathrm{out}|^2}{|s_\mathrm{in}|^2} = c^2+\frac{4\kappa_\mathrm{in}\kappa_\mathrm{out}}{\kappa^2}\frac{1}{1+u^2}-\frac{2c\sqrt{\kappa_\mathrm{in}\kappa_\mathrm{out}}}{\kappa}\frac{e^{i\varphi}(1-iu)+e^{-i\varphi}(1+iu)}{1+u^2}. \quad \text{(S10)}$$

Here we defined $u \equiv 2(\Delta-\delta\omega_\mathrm{c}(t))/\kappa$, which implies u is detuning- and time-dependent. We now summarize equation (S10) as $R'(u)$ and find the Taylor expansion for small $\delta\omega_\mathrm{c}$ around $u_0 \equiv 2\Delta/\kappa$:

$$R'(u) = R'(u_0)-\frac{2\delta\omega_\mathrm{c}}{\kappa}\frac{\partial R'(u_0)}{\partial u}+\ldots+\frac{(-2\delta\omega_\mathrm{c}/\kappa)^n}{n!}\frac{\partial^n R'(u_0)}{\partial u^n}, \quad \text{(S11)}$$

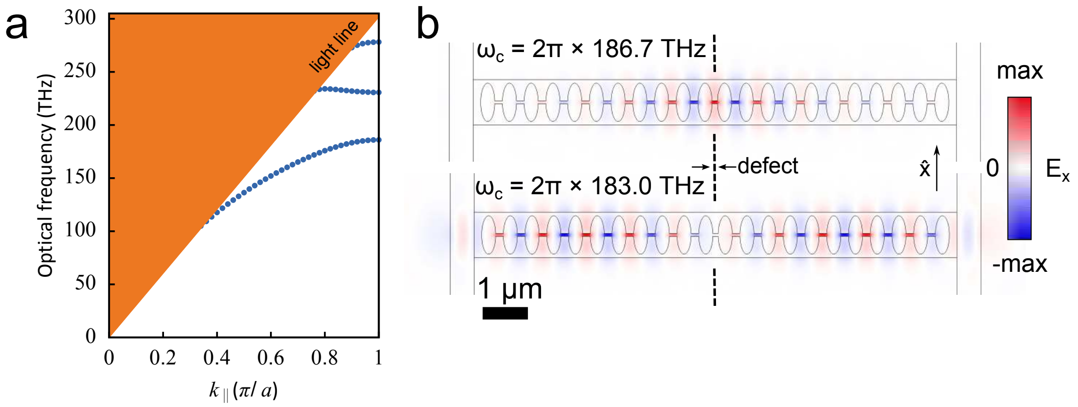

Figure S1. Optical modes in the sliced nanobeam. (a) Simulated dispersion diagram showing the TE-like waveguide modes in the periodic part of the sliced nanobeam. A bandgap is opened by the periodic structure of elliptical holes around a frequency of 200 THz. (b) Simulated transverse electric field profiles of the first two cavity modes of the structure. The defect that is responsible for the creation of these modes is a slightly smaller distance between the two holes in the center of the beam.

where the last term depicts the nth order in the Taylor expansion. We take a harmonic modulation of the cavity frequency $\delta\omega_c(t) = A\cos\Omega t$. To leading order, $\delta\omega_c^n \approx A^n \cos(n\Omega t)/2^{n-1}$. This means that each successive term in the Taylor expansion in equation (S11) gives the amplitude of a term at different frequency.

In the optomechanical system, the variance of the frequency modulations at the mechanical frequency Ω_m is given by $\langle\delta\omega_c^2\rangle = G^2\langle x^2\rangle_{th} = 2g_0^2 k_B T/\hbar\Omega_m$. Therefore we get the same variance in R if we set $A = 2g_0\sqrt{k_B T/\hbar\Omega_m}$. We note that $\frac{\partial^n R'(u_0)}{\partial u^n} = (\kappa/2)^n \frac{\partial^n R(\Delta)}{\partial\Delta^n}$, which for the variance of the signal at $n\Omega_m$ leads to

$$\left.\frac{\langle P^2\rangle}{P_{in}^2}\right|_{n\Omega_m} = \langle R^2\rangle\Big|_{n\Omega_m} = \frac{2(g_0^2 k_B T/\hbar\Omega_m)^n}{n!^2}\left(\frac{\partial^n R(\Delta)}{\partial\Delta^n}\right)^2. \tag{S12}$$

For $n = 1$, the result of equation (3) in the main text is again reproduced. The result of a calculation of the variances of $n = 1$–4, for the parameters used in the experiment, is shown in the main text in Fig. 5c.

Waveguide modes in the sliced nanobeam

In this section we discuss the waveguide modes in the periodic region of the sliced nanobeam in more detail. The free-standing silicon nanobeam acts as a waveguide, which can guide light via total internal reflection. In this waveguide, the elliptical holes form a photonic crystal that opens a bandgap for modes with transverse electric (TE)-like symmetry (Fig. S1a). This is not a full bandgap, since TM-like waveguide modes exist in the gap region. If the symmetry of the structure is broken by fabrication imperfections, light in the bandgap region for TE-like modes can scatter to the TM-like modes and propagate along the nanobeam. For this reason, this is sometimes referred to as a quasi-bandgap.

The guided mode at the lower edge of the band gap has the largest concentration of energy in the nanoscale gap in the middle of the beam. The fact that a significant portion of this mode's energy is located in vacuum increases its frequency in comparison to a non-sliced nanobeam, which reduces the frequency width of the bandgap. To ensure maximum mirror strength, the transverse size of the holes is made as large as possible. The elliptical hole shape is as such important to realize a strong bandgap, in addition to providing favorable mechanical properties as mentioned in the main text.

To create optical cavity modes that are derived from the lower band edge, the defect is a local decrease in distance between two elliptical holes, which decreases the local effective refractive index and creates defect states in the bandgap region. Fig. S1b shows the first two cavity modes created in this way in the sliced nanobeam. Note that the higher-order cavity modes have a lower frequency, since they are less confined near the defect, so that the frequency is closer to that of the waveguide mode in the periodic structure.

Overlap of optical and mechanical mode profiles

The frequency shift of the optical resonance of the sliced nanobeam due to mechanical motion depends on the overlap between the optical and mechanical mode profiles. We simulated the frequency shift of the optical resonance of the sliced nanobeam due to a uniform mechanical shift of 1 nm, as shown in the main text in Fig. 1d. To estimate the influence of the finite extent

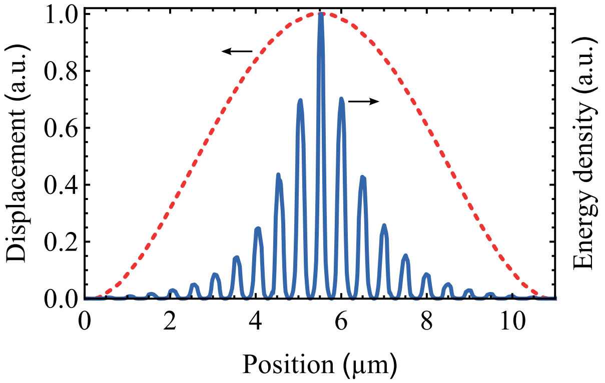

Figure S2. Normalized resonant mode profiles of the fundamental optical and mechanical resonance of the sliced nanobeam. The red dotted line shows the displacement along the length of the beam, while the blue solid line represents the local energy density.

of the mode profiles on the response, we extract these profiles from the numerical simulation. Fig. S2 shows the normalized displacement profile of the mechanical resonance and the normalized electromagnetic energy density profile of the optical cavity mode, as a function of the position along the nanobeam.

The mechanical mode profile closely resembles the mode profile of the fundamental mode of a doubly-clamped beam. From the simulated displacement profile we calculate the effective mass for this purely antisymmetric motion to be $m_{\mathrm{eff}} \approx 0.39m$, where m is the total mass of the sliced nanobeam, which is indeed very close to the value obtained from the analytical displacement profile of a uniform doubly-clamped beam.[3]

The optical mode profile clearly shows the localization of the energy density in the small gaps between the silicon 'teeth' of the structure. The simple defect we introduce in the center of the beam localizes the optical cavity mode there, while the field decays exponentially away from the defect, where the optical frequency lies inside the photonic quasi-bandgap.

We compute the correction on the frequency shift due to the finite extent of the modes from the overlap integral between these two mode profiles, and find a factor of 0.90 with respect to the frequency shift for a uniform displacement of the beam.

The higher-order cavity modes created by the defect are less strongly confined along the length of the beam, as shown in Fig. S1b. Since the fundamental mechanical resonance has the largest displacement in the center of the beam, the higher-order cavity modes are less sensitive to this motion than the fundamental optical resonance.

Mechanical mode coupling

The mechanical modes observed in a two-beam system are combinations of the motion of the individual beams, with in the perfectly symmetrical case fully in-phase or out-of-phase motion. Here we derive the consequences of imperfect symmetry for the ratio of scattered power between the two modes.[4]

Harmonic motion of the two beams at a certain frequency Ω can be described as:

$$\begin{bmatrix} x_1(t) \\ x_2(t) \end{bmatrix} = \begin{bmatrix} \psi_1 \\ \psi_2 \end{bmatrix} \cos\Omega t \equiv \vec{\psi}\cos\Omega t, \text{ where } \vec{\psi} = \begin{bmatrix} \psi_1 \\ \psi_2 \end{bmatrix}. \tag{S13}$$

Thus, ψ_1 and ψ_2 are the amplitudes of the oscillatory motion of the two individual beams, such that their variance is $\langle \psi_{1,2}^2 \rangle = \frac{1}{2}\psi_{1,2}^2$.

The optical response of the system is determined by the change in the distance between the beams: $d = x_1 - x_2$. Here we define x as $x \equiv d/2$, which leads to the same value of $G = \partial\omega_c/\partial x$ for both mechanical modes. Note that this choice of x corresponds to the lab-frame displacement of the two beams if they move in antiphase. The variance of x due to harmonic motion described by $\vec{\psi}$ is then:

$$\langle x^2 \rangle_\psi = \frac{1}{8}\left(\psi_1^2 + \psi_2^2 - 2\psi_1\psi_2\right). \tag{S14}$$

The state vectors of the two normal modes can be written without loss of generality as

$$\vec{\psi}_\alpha = A_\alpha \begin{bmatrix} \cos\theta \\ \sin\theta \end{bmatrix}, \quad \vec{\psi}_\beta = A_\beta \begin{bmatrix} \sin\theta \\ -\cos\theta \end{bmatrix}. \tag{S15}$$

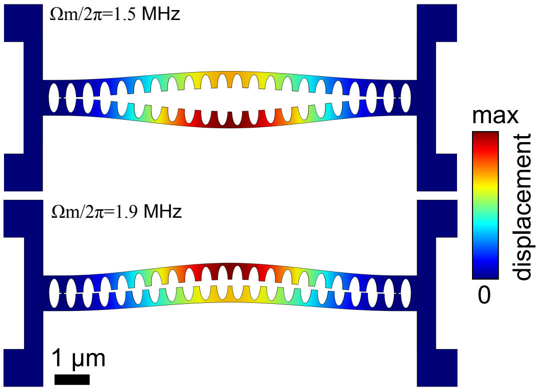

Figure S3. Simulated mechanical displacement profiles of the two fundamental in-plane resonances, including disorder and compressive stress. The dimensions of the beam were matched to the realized dimensions using measurements with a scanning electron microscope, including differences in hole and gap size along the beam. Compressive stress was introduced in the simulation by displacing one of the support pads by 10 nm along the direction of the beam.

For both of these modes, we can calculate the variance of x, denoted as $\langle x^2\rangle_\alpha$ and $\langle x^2\rangle_\beta$, respectively:

$$\begin{aligned}\langle x^2\rangle_\alpha &= \frac{1}{8}A_\alpha^2(\cos^2\theta+\sin^2\theta+2\sin\theta\cos\theta)\\ &= \frac{A_\alpha^2}{8}(1+\sin 2\theta),\\ \langle x^2\rangle_\beta &= \frac{A_\beta^2}{8}(1-\sin 2\theta)\end{aligned} \tag{S16}$$

For the beams undergoing thermal motion, the variance is given by the equipartition theorem:

$$\langle x^2\rangle_\alpha = \frac{k_B T}{m_\alpha \Omega_\alpha^2}, \quad \langle x^2\rangle_\beta = \frac{k_B T}{m_\beta \Omega_\beta^2}, \tag{S17}$$

where m_α and m_β are the effective mass of these modes. As shown in the previous section, for the differential mode the simulated effective mass with respect to the displacement coordinate x is $m_{\mathrm{eff}} = 0.39m$, with m the total mass of the sliced nanobeam. Evaluating equations (S16) and (S17) for a differential mode ($\theta = \pi/4$) yields $A_\alpha^2 = 4k_BT/m_{\mathrm{eff}}\Omega_\alpha^2$ and similarly for A_β^2. Substituting this back into equation (S16), we arrive at

$$\langle x^2\rangle_\alpha = \frac{k_BT(1+\sin 2\theta)}{m_{\mathrm{eff}}\Omega_\alpha^2}, \quad \langle x^2\rangle_\beta = \frac{k_BT(1-\sin 2\theta)}{m_{\mathrm{eff}}\Omega_\beta^2}. \tag{S18}$$

We note that thermal variance is related to the zero-point fluctuations x_{zpf} as

$$\langle x^2\rangle_\psi = 2\frac{k_BT}{\hbar\Omega_\psi}(x_{\mathrm{zpf}}^\psi)^2, \text{ so } x_{\mathrm{zpf}}^\psi = \sqrt{\frac{\hbar(1\pm\sin 2\theta)}{4m_{\mathrm{eff}}\Omega_\psi}}, \tag{S19}$$

where $\psi = \alpha, \beta$ and $+$ respectively $-$ is chosen as the sign for the term $\sin 2\theta$.

Finally, we measure $\langle P^2\rangle$ and wish to relate this to a displacement variance $\langle x^2\rangle$. We calculated m_{eff} using the simulated mode profile and assume the thermal bath temperature of the mechanical modes T is equal to the lab temperature, which leaves only θ and the transduction factor $\partial P/\partial x$ unknown. By measuring the area of both peaks $\langle P^2\rangle_\alpha$ and $\langle P^2\rangle_\beta$, we resolve the remaining ambiguity, allowing us to calibrate the displacement spectrum.

Influence of compressive stress and experimental disorder

The simulation of the ideal structure shown in the main text predicts the resonance frequency of the fundamental in-plane resonance to be 6 MHz, and additionally the frequency difference between the anti-symmetric and symmetric mode is negligible.

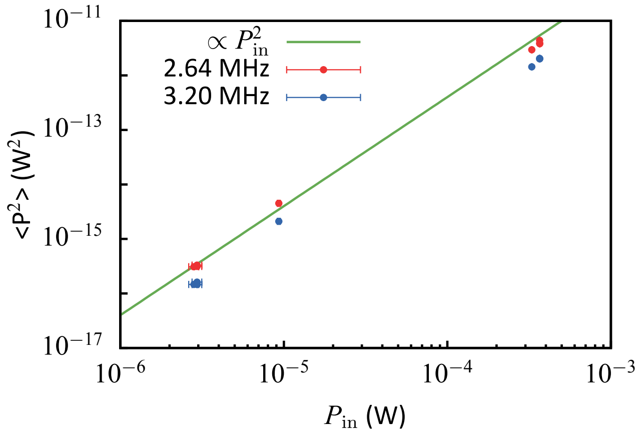

Figure S4. Power dependence. The red and blue datapoints show the measured signal from the two fundamental mechanical resonances, obtained by fitting both mechanical resonances in the spectra (see Fig. 2 in the main text for such a spectrum). The data was taken at various input powers with resonant laser light (zero detuning). The green line is a guide to the eye, with a slope corresponding to quadratic dependence on input power. The errorbars indicate readout error of the input power but don't take into account possible variations in incoupling efficiency due to slight changes in alignment.

We expect the frequency of the out-of-plane resonances to be larger than that of the fundamental in-plane mode, as the narrowest part of the half-beams (80 nm) is smaller than the Si slab thickness (200 nm).

In our experiment we find significantly smaller values of 2.6 and 3.2 MHz for one structure, and 1.4 and 2.0 MHz for a second structure. The differences between the frequencies of the two in-plane resonances are also larger than expected. To find the origin of this effect we measure the experimentally observed disorder produced by the fabrication process of the second structure using a scanning electron microscope. We then introduce these dimensions into the simulation, which results in slightly different eigenfrequencies. However, the resulting values are still near 6 MHz and additionally the two fundamental modes still have only a small frequency difference.

Finally, we add an extra step to the simulation to include a static compressive stress, which can result from the stress in the buried oxide layer of the silicon-on-insulator layer structure we use.[5] We define an initial displacement for one of the support pads along the length of the beam, which creates the stress. Increasing the amount of displacement, and therefore the stress, decreases the eigenfrequencies predicted by the simulation. This is in accordance with the theoretical expectation.[6] At the same time the difference between the eigenfrequencies of the two fundamental modes increases.

Fig. S3 shows the displacement profiles that result from such a simulation. At a displacement value of 10 nm (0.09% of the length of the beam), the resonances occur at frequencies very close to the experimentally measured values, at 1.5 and 1.9 MHz.

Influence of optical input power

In the measurements shown in the main text, we use up to 370 μW of optical power incident on the nanobeam. Using the parameters of our fit to the reflection spectrum, we estimate that this results in an intracavity intensity that corresponds to a maximum of $\approx$ 1000 photons. For this intensity in the cavity, we do not expect an increase in the cavity temperature of more than a few Kelvin. As a first confirmation of this, we see thermal shifts of the cavity resonance frequency of less than 1 nm, which corresponds to a temperature increase of less than 10%.

To check the assumption more thoroughly we measured the signal strength of the two fundamental mechanical frequencies as a function of power incident on the structure. Fig. S4 shows the result for resonant light, both from the zero-detuning point in swept measurements and from individual measurements where the detuning was set to zero by minimizing the optically induced shift of the mechanical frequency. Comparison with the line shows that the datapoints closely follow the expected quadratic dependence on input power for both peaks in the spectrum. The largest source of uncertainty in this measurement are differences in coupling efficiency between the incoming laser beam and the cavity due to small changes in alignments, which can influence the signal strength between measurements.

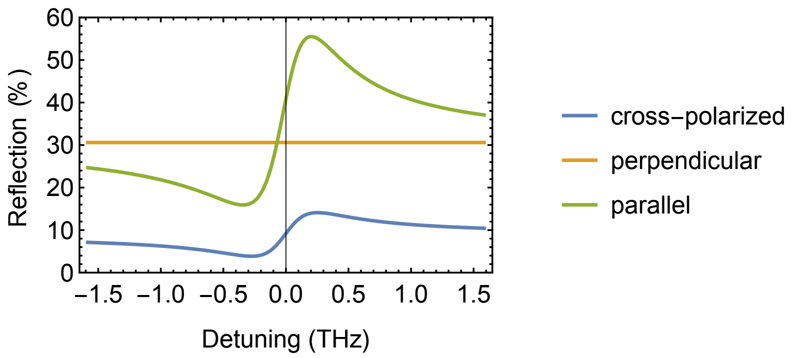

Figure S5. Calculated reflection curves for various polarization settings: cross-polarized (bottom, blue curve), with the cavity at 45° to both, and with input and output polarizers both oriented perpendicular and parallel to the cavity polarizability (middle (flat) orange curve and top green curve, respectively).

Cross-polarized reflection

In the main text we show only cross-polarized reflection spectra, with the cavity oriented at 45° with respect to both the input and output polarization. Our reflection setup does not allow measuring non-cross-polarized reflection directly due to our use of a polarizing beamsplitter. However, we can simulate what we expect for the reflection based on a simplified model of the experiment. For this we assume the coupling to the cavity, specifically its polarization response, can be described as coupling to a dipole polarizability. This means it will not couple at all when light is polarized perpendicular to the polarizability and the coupling will be exactly $1/2$ when oriented at 45°. Additionally, we need to estimate the direct reflection when not using a cross-polarization scheme. The main contribution to this is the reflection of the flat silicon substrate interface, which we calculate using the Fresnel equations. This yields a reflectivity of 31%. The additional contribution of non-resonant scattering from the nanobeams as well as a possible contribution from the (unpolished) back surface of the silicon substrate have been neglected. Fig. S5 shows the result of this calculation, using the fitted parameters from the reflection spectrum shown in Fig. 3a in the main text.

In our experiment, we also inserted a quarter-wave plate between the polarizing beamsplitter and the sample. By turning the quarter-wave plate, we could tune the incident and outgoing polarization between linear and circular, simultaneously varying the degree of suppression of the direct reflection. We verified that this yielded the expected results: first a large effect on the shape of the resulting Fano lineshape, due to the amplitude change of the direct reflection as well as the phase delay introduced by the slow axis of the waveplate; second a relatively small effect on the rate of coupling to the cavity, since the rate of coupling to a dipolar resonator hardly changes when going from linear at 45° to circular polarization. We note that for some settings of the waveplate, it was possible to reach an almost perfect Lorentzian peak or dip in the reflection spectrum, which suggests the contribution of non-resonant scattering of the nanobeam can interfere destructively or constructively with direct reflection from the silicon substrate at these settings.

References

1. Gorodetsky, M. L., Schliesser, A., Anetsberger, G., Deleglise, S. & Kippenberg, T. J. Determination of the vacuum optomechanical coupling rate using frequency noise calibration. *Opt. Express* **18**, 23236–46 (2010).

2. Doolin, C. *et al.* Nonlinear optomechanics in the stationary regime. *Phys. Rev. A* **89**, 053838 (2014).

3. Cleland, A. N. *Foundations of Nanomechanics* (Springer-Verlag Berlin Heidelberg, 2003).

4. Thijssen, R., Kippenberg, T. J., Polman, A. & Verhagen, E. Plasmomechanical Resonators Based on Dimer Nanoantennas. *Nano Lett.* (2015).

5. Yamashita, D., Takahashi, Y., Asano, T. & Noda, S. Raman shift and strain effect in high-Q photonic crystal silicon nanocavity. *Opt. Express* **23**, 3951 (2015).

6. Bokaian, A. Natural frequencies of beams under compressive axial loads. *J. Sound Vib.* **126**, 49–65 (1988).
